# Compound‐specific isotope analysis of benthic foraminifer amino acids suggests microhabitat variability in rocky‐shore environments

**DOI:** 10.1002/ece3.4358

**Published:** 2018-07-24

**Authors:** Masashi Tsuchiya, Yoshito Chikaraishi, Hidetaka Nomaki, Yoko Sasaki, Akihiro Tame, Katsuyuki Uematsu, Naohiko Ohkouchi

**Affiliations:** ^1^ Japan Agency for Marine‐Earth Science and Technology Yokosuka Japan; ^2^ Institute of Low Temperature Science Hokkaido University Sapporo Japan; ^3^ Marine Works Japan, Ltd. Yokosuka Japan

**Keywords:** habitat segregation, molecular characterization, nitrogen isotopic composition of amino acids, rocky‐shore benthic foraminifera, trophic position

## Abstract

The abundance and biomass of benthic foraminifera are high in intertidal rocky‐shore habitats. However, the availability of food to support their high biomass has been poorly studied in these habitats compared to those at seafloor covered by sediments. Previous field and laboratory observations have suggested that there is diversity in the food preferences and modes of life among rocky‐shore benthic foraminifera. In this study, we used the stable nitrogen isotopic composition of amino acids to estimate the trophic position, trophic niche, and feeding strategy of individual foraminifera species. We also characterized the configuration and structure of the endobiotic microalgae in foraminifera using transmission electron microscopy, and we identified the origin of endobionts based on nucleotide sequences. Our results demonstrated a large variation in the trophic positions of different foraminifera from the same habitat, a reflection of endobiotic features and the different modes of life and food preferences of the foraminifera. Foraminifera did not rely solely on exogenous food sources. Some species effectively used organic matter derived from endobionts in the cell cytoplasm. The high biomass and species density of benthic foraminifera found in intertidal rocky‐shore habitats are thus probably maintained by the use of multiple nitrogen resources and by microhabitat segregation among species as a consequence.

## 1 INTRODUCTION

The study of environmental adaptation of organisms is essential to provide accuracy in our understanding of the distribution, ecology, population dynamics, species diversity, and evolution of the organisms. The benthic foraminifera are unicellular eukaryotic microorganisms that live under a wide range of environmental conditions through almost entire seafloor and even in the freshwater sediments. The wide range of foraminiferal distributions reflects a variety of environmental conditions that differ in terms of redox conditions and the quantity and type of organic matter (e.g., Corliss, 1985; Kitazato, 1988; Langer, 1993; Levin et al., 1999; Lipps & Valentine, 1970; Nomaki, Heinz, Nakatsuka, Shimanaga, & Kitazato, 2005; Nomaki et al., 2006; Ohga & Kitazato, 1997; Silva, Corliss, Rathburn, & Thunell, 1995). Furthermore, foraminifera can adapt to a wide range of environmental conditions, with or without ecto/endobiotic bacteria (e.g., Bernhard, Buck, & Barry, 2001; Bernhard, Buck, Farmer, & Bowser, 2000; Bernhard, Tsuchiya, & Nomaki, 2018; Risgaard‐Petersen et al., 2006). The study of foraminifera in these diverse conditions thus can provide valuable insights on the environmental adaptation and evolution of aquatic organisms. However, the mechanisms that underlie adaptation of foraminifera to microhabitats, nutritional strategies, and interspecific and intraspecific competition for resources have not yet been fully resolved, particularly with respect to the trophic function and the dynamics within the lower‐trophic‐level hierarchy of ecosystems.

It is well known that intertidal rocky‐shore environments have relatively high species diversity (e.g., Lewis, 1964; Suchanek, 1992). This environment exhibits complex microhabitats among the irregularities of rocks and seaweeds, with foraminiferal diversity and biomass being high in the algal microhabitat (e.g., Kitazato, 1986, 1988). Seawater is well mixed at intertidal rocky shores because of the high‐energy nature of the environment, and dissolved oxygen and available foods (as organic resources) are therefore supplied in sufficient amounts to the microhabitats. Unlike sediments, no obvious environmental gradients are therefore found in these factors. In contrast, the large biomass of seaweeds and microtopographic features of intertidal rocky shores lead to a large variation in the level of sunlight and hence in the biomass of photoautotrophic epiphytic microalgae that are potential foods for foraminifera. It is thus possible that the rocky‐shore foraminiferal community in algal microhabitats is structured by factors that differ from those that structure benthic foraminiferal communities in sediments, that is, redox conditions and food supplies. Indeed, factors that control the high density and trophic niches of foraminifera in algal microhabitats have not yet been investigated in rocky‐shore environments. Moreover, considering that some rocky‐shore benthic foraminifera possess photoautotrophic endobionts or kleptoplasts, it is likely that the trophic requirements of shallow‐water foraminifera for particular organic and inorganic substrates depend on the presence or absence of endobionts, the endobiont type (species), and the form of the endobiont (microalgae or kleptoplast) (e.g., Lee et al., 1991).

Several previous studies have investigated the adaptation and habitat preference of foraminifera in algal microhabitats based on field and laboratory feeding experiments in rocky shores (e.g., Kitazato, 1988; Langer, 1993). Those studies have identified four modes of life (based on attached substrates and way of locomotion) in the microhabitats of coralline algae and the associated trapped detrital sediments: phytal (living between the coralline algal stems), crawling (attached, mobile on the thalli of coralline algae), attached (immobile, fixed on both coralline algae and substrate), and free‐living forms (living in and on the sediment, as well as on the coralline algae) (Kitazato, 1988, 1994). These different modes of life were suggested to be related to different food requirements; however, it is also unclear to what extent foraminiferal food requirements are related to their mode of life.

In particular, although many foraminiferal species retain kleptoplasts (i.e., sequestered chloroplasts) that could be used for photosynthesis, photosynthesis using those kleptoplasts has not yet been shown to be a direct source of their nutrition. If foraminifera do not require an exogenous food source but instead depend on endobionts for their food, it would be unnecessary for them to capture food. On the other hand, the fact that they maintain a high individual density in a microhabitat may compromise their ability to compete for food, not only among foraminiferal species but also between other heterotrophic organisms. Thus, accurate estimate of foraminiferal trophic position, including species having kleptoplast or algal endobionts, is required for better understanding on trophic niche separation at rocky‐shore environments.

Trophic position (TP) can be estimated based on the nitrogen isotopic composition of glutamic acid (*δ* ^15^N_Glu_) and phenylalanine (*δ* ^15^N_Phe_) generally with an error of 0.1–0.2 units (Chikaraishi, Ogawa, & Ohkouchi, 2010; Chikaraishi et al., 2009, 2014). It is possible to estimate the TP with the difference between *δ* ^15^N_Glu_ and *δ* ^15^N_Phe_ values, because the former amino acid shows a large *δ* ^15^N enrichment from one trophic level to the next, whereas the latter shows little change in the *δ* ^15^N value between trophic levels. Each amino acid evidences different isotopic fractionation during amino acid metabolism. Use of the *δ* ^15^N of these two amino acids is a powerful tool for elucidating the trophic position of organisms in aquatic ecosystems (Chikaraishi et al., 2009). During the last four decades, the empirical enrichments of ^13^C and ^15^N in bulk organic tissues from prey to predator species (~0.8‰ for ^13^C and ~3.4‰ for ^15^N, DeNiro & Epstein, 1978; Minagawa & Wada, 1984) have been used for estimating prey‐predator relationships. However, when being used to estimate the trophic position of a certain organism, the traditional bulk method is hampered by the spatial and temporal variation in the *δ* ^15^N value of primary producers. As a consequence, the uncertainty of the estimated trophic position is often too large to provide detailed information about the diet of the organism. In contrast, an estimate of trophic position based on the *δ* ^15^N values of trophic and source amino acids (i.e., glutamic acid and phenylalanine, respectively) from a single organism is independent of such factors. The result is that the trophic positions of organisms and their use of resources can be better estimated (Chikaraishi et al., 2014; Ohkouchi, Ogawa, Chikaraishi, Tanaka, & Wada, 2015). Many studies have used this compound‐specific isotopic analysis technique to successfully address a wide range of topics, including assessment of trophic position in various ecosystems, reconstruction of animal migration and environmental variability, and assessment of marine organic matter dynamics (reviewed in Ohkouchi et al., 2017).

In this study, we applied this method to foraminifera to estimate of trophic hierarchy to rocky‐shore benthic foraminifera, with an emphasis on whether foraminifera gain nutrition through endobionts in their cell cytoplasm and to illustrate above‐mentioned diverse trophic ecology in their rocky‐shore habitats. We estimated the trophic position of seven dominant species of rocky‐shore benthic foraminifera, including all four different modes of life. We also conducted ultrastructural observations by transmission electron microscopy (TEM) to clarify the structures and configurations of endobionts, and we performed molecular characterizations via nucleotide sequencing to determine the origin of the endobionts. From these results, we then discuss the trophic hierarchy among species of foraminifera with respect to their modes of life, food preferences, and the nutritional relationships between algal endobitons or kleptoplasts.

## 2 MATERIALS AND METHODS

### 2.1 Sample collection

We collected specimens of benthic foraminifera that are commonly found in the intertidal rocky‐shore environment along the coast of Japan. These benthic foraminifera live in the microhabitats of coralline algae, from which we selected a total of seven species associated with the four main modes of life (Kitazato, 1988)—the phytal form: *Pararotalia nipponica* Asano, *Elphidium* aff. *E*. *crispum* (Linnaeus); the crawling form: three glabratellid species including *Planoglabratella opercularis* (d'Orbigny), *Glabratella patelliformis* (Brady), and *Angulodiscorbis quadrangularis* Uchio; the attached form: *Cibicides lobatulus* (Walker and Jacob); and the free‐living form: *Quinqueloculina yabei* Asano (Table 1). For the compound‐specific stable nitrogen isotope analysis of amino acids, we used 10–200 individual foraminiferal specimens for amino acid extraction depending on their cell size, because the amounts of glutamic acid and phenylalanine were too small to detect within a single specimen. These seven species account for 70%–80% of the foraminiferal assemblages in rocky‐shore environments along the Japanese coast (Kitazato, 1988) and are representative of foraminiferal assemblages that occupy microhabitats. We used open nomenclature for *E*. aff. *E*. *crispum* in accord with Jauffrais et al. (2018) because our nucleotide sequence data are different from the European *E*. *crispum* phylotype S11 (Darling et al., 2016). However, the previous studies have characterized Japanese specimens as *E. crispum*. To avoid confusion, we use *E*. *crispum* hereafter for this species.

**Table 1:** Foraminifer sampling locations, ecology, and sample preparation for nitrogen isotopic analysis of amino acids

Species	Sample ID	Sampling site	Condition	Sampling date	Type of symbiosis^a^	Origin of symbiont^b^	Test type^c^	Microhabitat in coralline algae^d^	Mode of life^e^	H_2_O_2_ treatment^f^	Analyzed part^g^	Number of individual specimens
*P*. *opercularis*	160108‐3	Omaezaki, Shizuoka	Nature (Day)	21 April 2015	K	B	Hyaline, High Mg	F	C	−	Cell	200
	111205‐4	Minami‐izu, Shizuoka	Nature (Day)	13 July 2011						−	Cell	25
	120607‐1	Yugawara, Kanagawa	Nature (Day)	8 May 2012						+	Test	122
	120607‐2									−	Cell	122
	160118‐2									−	Cell	35
*G*. *patelliformis*	120606‐7	Yugawara, Kanagawa	Nature (Day)	8 May 2012	K	B	Hyaline, High Mg	F	C	+	Test	69
*A*. *quadrangularis*	120607‐3	Yugawara, Kanagawa	Nature (Day)	8 May 2012	K	B	Hyaline, High Mg	F	C	+	Test	80
	120607‐4									−	Cell	79
	160112‐2			20 April 2015						−	Cell	42
	160118‐3		Nature (Night)	19 April 2015						−	Cell	88
*E*. *crispum*	120606‐1	Yugawara, Kanagawa	Nature (Day)	8 May 2012	K	B	Hyaline, High Mg	F	P	+	Test	131
	120606‐2									−	Cell	131
	160112‐3			20 April 2015						−	Cell	100
	160118‐4		Nature (Night)	19 April 2015						−	Cell	100
*P*. *nipponica*	160108‐4	Omaezaki, Shizuoka	Nature (Day)	21 April 2015	*	B	Hyaline, Low Mg	F	P	−	Cell	200
	120606‐5	Yugawara, Kanagawa	Nature (Day)	8 May 2012						+	Test	90
	120606‐6									−	Cell	90
*C*. *lobatulus*	160112‐1	Omaezaki, Shizuoka	Nature (Day)	21 April 2015	None	‐	Hyaline, Low Mg	F, S, T	A	−	Cell	95
	120606‐3	Yugawara, Kanagawa	Nature (Day)	8 May 2012						+	Test	99
	120606‐4									−	Cell	98
	160112‐4			20 April 2015						−	Cell	58
	160126‐1		Nature (Night)	19 April 2015						−	Cell	60
*Q*. *yabei*	120607‐5	Yugawara, Kanagawa	Nature (Day)	8 May 2012	None	‐	Porcellaneous, High Mg	S, T, Fl	F	+	Test	11
	120607‐6									−	Cell	10
	160118‐1			20 April 2015						−	Cell	27
	160126‐2		Nature (Night)	19 April 2015						−	Cell	42

*Notes*

Shading indicates sampling site and period; white: collected from Omaezaki or Minami‐Izu in the daytime, gray: collected from Yugawara in the daytime, dark gray: collected from Yugawara at night.

^a^K, kleptoplast;^b^B, Bacillariophyta (diatom). ^c^High Mg, high magnesian calcite; low Mg, low magnesian calcite (from Toyofuku, Kitazato, Kawahata, Tsuchiya, & Nohara, 2000; Toyofuku et al., 2011). ^d^F, frond; S, stem; T, thallus; Fl, flocculent layer. Classification of foraminiferal microhabitats in coralline algae from Kitazato (1988). ^e^C, crawling form; P, phytal form; A, attached form; F, free living. Rocky‐shore foraminiferal modes of life were from Kitazato (1988). ^f^+, with H_2_O_2_ treatment—measures the nitrogen isotopic composition of organic membranes within the calcareous shell (intracrystalline protein); −, without H_2_O_2_ treatment—measures the nitrogen isotopic composition of both cytoplasm and intracrystalline proteins. ^g^Test, organic matters including both organic sheet and intracrystalline protein in the test; cell, organic matter in the test and cytoplasm.

*Endobiotic algae.

The specimens were collected from the upper to lower intertidal zone of a rocky‐shore environment at Yugawara, Kanagawa Prefecture (35°08.9ʹN, 139°07.5ʹE) in 2012 and 2015. Specimens of *P*. *opercularis* were also collected from Minami‐Izu, Shizuoka Prefecture (34°36.9ʹN, 138°49.3ʹE) in 2011, and additional specimens of *P*. *opercularis*,* P*. *nipponica*, and *C*. *lobatulus* were collected from Omaezaki‐Cape, Shizuoka Prefecture (34°35.6ʹN 138°13.6ʹE) in 2015 (Table 1; Supporting information Figure S1). To investigate trophic adaptability, these samples (except for *G*. *patelliformis*) were collected from multiple localities on 3–5 different occasions during different weather conditions and during both day and night (i.e., different levels of irradiance). Irradiance levels (μmol photon m^−2^ s^−1^ PAR of 400–700 nm radiation) under water (in the air) included 0 (0) and 791 (1,568) at Yugawara at night and during the day in 2015, respectively, and 1,850 (2,570) at Omaezaki during the day in 2015. The environmental conditions at the sites were very similar except temperature as follows—temperature: 16.0°C, salinity: 34.4, dissolved oxygen (DO) concentration: 12.08 mg/L, *p*H: 8.13 in the daytime; temperature: 15.6°C, salinity: 34.2, DO: 9.17 mg/L, *p*H: 7.81 at night in Yugawara; temperature: 18.4°C, salinity: 34.3, DO: 12.1 mg/L, *p*H: 8.29 in Omaezaki; and temperature: 27.0°C, salinity: 24.3, DO: 12.9 mg/L, *p*H: 8.25 in Minami‐Izu. The foraminifera were found on the coralline alga *Corallina pilulifera* (Rhodophyta) and in the detrital sediment trapped within the alga. They were collected from a water depth of 1 m under full sunlight in the summer at the Minami‐Izu site, in clear weather at Omaezaki, and in overcast weather at Yugawara. The Yugawara site was located at the foot of a bridge where the direct sunlight was blocked at all times; rhodophytes, mainly coralline algae, flourished at this site, despite the shallow‐water depth.

To analyze the impact of light on the foraminiferal nutritional conditions, it was necessary to compare different levels of irradiance. Photosynthetic reactions occur even at low irradiances. We compared foraminiferal responses to the effects on endobiont photosynthesis for at least three irradiances.

### 2.2 Amino acid nitrogen isotope analysis and estimation of trophic hierarchy

Living individual foraminifera were retrieved using a Pasteur pipette under a binocular microscope just after sample collection. The Yugawara and Omaezaki samples were immediately frozen at −20°C or with dry ice to avoid effects of endobiotic photosynthesis and digestion of food materials in foraminiferal cell. These samples were either treated or nor with hydrogen peroxide to obtain the isotopic composition of the intracrystalline protein in the test (shell) or of the bulk cell (sum of cell cytoplasm, organic membranes, and intracrystalline protein), respectively (Table 1). Foraminiferal organic matter exists not only in the cell, but also in the intracrystalline proteins in the test that act as a template for calcium carbonate shell growth. If shell growth occurred under different environmental conditions, large differences would be expected between cytoplasm and intracrystalline proteins. Thus, the nitrogen isotope values in intracrystalline proteins would be expected to suggest long‐term values, whereas that in the bulk cells would be expected to indicate the short‐term value depends on their metabolism.

The isotopic composition of amino acids was determined according to the method of Chikaraishi et al. (2009). Each specimen was hydrolyzed in 12 M HCl at 110°C, and then, the hydrolyzate was washed with *n*‐hexane/dichloromethane (3:2, v/v) to remove any hydrophobic constituents. After derivatization with thionyl chloride/2‐propanol (1:4, v/v) and subsequently with pivaloyl chloride/dichloromethane (1:4, v/v), the derivatives of the amino acids were extracted with *n*‐hexane/dichloromethane (3:2, v/v). The nitrogen isotopic composition of individual amino acids was determined by gas chromatography/combustion/isotope ratio mass spectrometry (GC/C/IRMS) using a Delta^plus^XP isotope ratio mass spectrometer (Thermo Fisher Scientific, Bremen, Germany) coupled with a 6890N gas chromatograph (Agilent Technologies, Santa Clara, CA, USA) via combustion and reduction furnaces. Nitrogen isotopic composition is expressed in conventional *δ*‐notation against the nitrogen isotopic composition of air.

The trophic position (TP_Glu/Phe_) of the sample was calculated based on the following equation proposed by Chikaraishi et al. (2009):

(1) $${\text{TP}}_{\mathrm{Glu}/\mathrm{Phe}}=\{\left(\delta^{15}N_{\mathrm{Glu}}-\delta^{15}N_{\mathrm{Phe}}-3.4)/7.6\right\}+1$$

where *δ* ^15^N_Glu_ and *δ* ^15^N_Phe_ represent the nitrogen isotopic composition of glutamic acid and phenylalanine, respectively, 3.4 is the isotopic difference between glutamic acid and phenylalanine in primary producers, and 7.6 is the offset of the ^15^N‐enrichment factor of these two amino acids per trophic position increase. Although this equation may need modification (particularly for the ^15^N‐enrichment factor) for some specific organisms, such modification is not required, at least for organisms in the lower‐trophic‐level hierarchy of food webs (e.g., McMahon & McCarthy, 2016). The trophic position is expected to be 1.0 for a “pure” primary producer and 2.0 for a “pure” primary consumer. The trophic position of a predator is 1 higher than the trophic position of its prey.

Previous studies have indicated that the potential uncertainty in the TP_Glu/Phe_ value calculated via propagation of error is 0.20–0.40 for each trophic level (Table 2) based on an assumed standard deviation of 0.5‰ (1*σ*) for the observed *δ* ^15^N values of glutamic acid and phenylalanine (Chikaraishi, Kashiyama, Ogawa, Kitazato, & Ohkouchi, 2007; Chikaraishi et al., 2009, 2014).

**Table 2:** Nitrogen isotopic composition of glutamic acid (*δ* ^15^N_Glu_) and phenylalanine (*δ* ^15^N _Phe_), and estimated trophic position (TP_Glu/Phe_) of rocky‐shore foraminifera

	Sample ID	*δ* ^15^N_Glu_ (‰)^a^	*δ* ^15^N_Phe_ (‰)^a^	TP_Glu/Phe_ b	Propagation error^c^
*P*.* opercularis*	160108‐3	7.8	2.7	1.2	0.35
	111205‐4	8.0	3.3	1.2	0.40
	120607‐1	16.6	5.7	2.0	0.22
	120607‐2	16.2	5.6	1.9	0.22
	160118‐2	11.8	2.0	1.8	0.23
*G*.* patelliformis*	120606‐7	14.4	3.4	2.0	0.22
*A*. *quadrangularis*	120607‐3	15.3	5.2	1.9	0.22
	120607‐4	15.7	5.9	1.8	0.23
	160112‐2	10.0	0.0	1.9	0.22
	160118‐3	11.1	2.7	1.7	0.24
*E*.* crispum*	120606‐1	9.9	4.6	1.2	0.34
	120606‐2	10.0	4.5	1.3	0.33
	160112‐3	12.0	4.1	1.6	0.25
	160118‐4	10.4	4.8	1.3	0.32
*P*. *nipponica*	160108‐4	11.2	‐0.9	2.2	0.21
	120606‐5	15.3	1.5	2.4	0.20
	120606‐6	16.2	1.9	2.4	0.20
*C*. *lobatulus*	160112‐1	10.8	‐0.9	2.1	0.21
	120606‐3	19.4	6.2	2.3	0.20
	120606‐4	19.3	5.5	2.4	0.20
	160112‐4	20.3	6.2	2.4	0.20
	160126‐1	16.4	3.3	2.3	0.20
*Q. yabei*	120607‐5	9.3	3.3	1.3	0.30
	120607‐6	10.5	4.9	1.3	0.32
	160118‐1	4.8	‐1.8	1.4	0.28
	160126‐2	8.3	1.1	1.5	0.26

*Notes*

Shading indicates sampling site and period; white: collected from Omaezaki or Minami‐Izu in the daytime, gray: collected from Yugawara in the daytime, dark gray: collected from Yugawara at night.

n.d., not determined.

^a^Relative to atmospheric nitrogen. ^b^TP_Glu/Phe_ = (*δ* ^15^N_Glu_ ‐ *δ* ^15^N_Phe_ ‐ 3.4)/7.6 + 1. ^c^Potential uncertainly in TP_Glu/Phe_ calculated with 1*σ* (0.5) for each value in equation.

### 2.3 Transmission electron microscopy observations

Transmission electron microscopy (TEM) observations were used to determine the presence or absence, and morphology of endobionts and the content of the food vacuole in the foraminiferal cell. Detailed procedures of preparation of samples for TEM have been provided by Jauffrais et al. (2018) and Tsuchiya et al. (2015). The TEM observation was carried out using a TECNAI G^2^ 20 transmission electron microscope (FEI, Hillsboro, OR, USA) at an acceleration voltage of 120 kV.

### 2.4 Molecular identification

DNA sequencing and homology searches were conducted to identify the origin of the endobionts. For host foraminiferal identification, we amplified the nuclear small subunit (SSU) ribosomal RNA (*rRNA*) gene to distinguish the host foraminifer with the primer pair of s14f1 and sB (Pawlowski, 2000). For endobiont discrimination, we amplified the plastid *16S rRNA* gene further to distinguish the origin of endobionts with primer pair PL16S1 and PL16S2 (Takishita, Nakano, & Uchida, 1999). All nucleotide sequences were checked to detect chimeric sequences using the Bellerophon server (Huber, Faulkner, & Hugenholtz, 2004) available online (http://comp-bio.anu.edu.au/bellerophon/bellerophon.pl, last accessed Jun 26, 2017); no chimeric sequences were found in this study. Detailed methods involved in individual sorting, PCR amplification, cloning, and sequencing have been described by Tsuchiya et al. (2009, 2015). We searched for the most similar sequences using the Basic Local Alignment Search Tool (BLAST) with homology search option (National Center for Biotechnology Information, National Institutes of Health, Bethesda, MD, USA). The sequences obtained in this study have been deposited in the GenBank database (accession numbers: KY498705–KY498734) (Supporting information Table S1).

## 3 RESULTS

### 3.1 Endobionts and their origins

**Figure 1:**
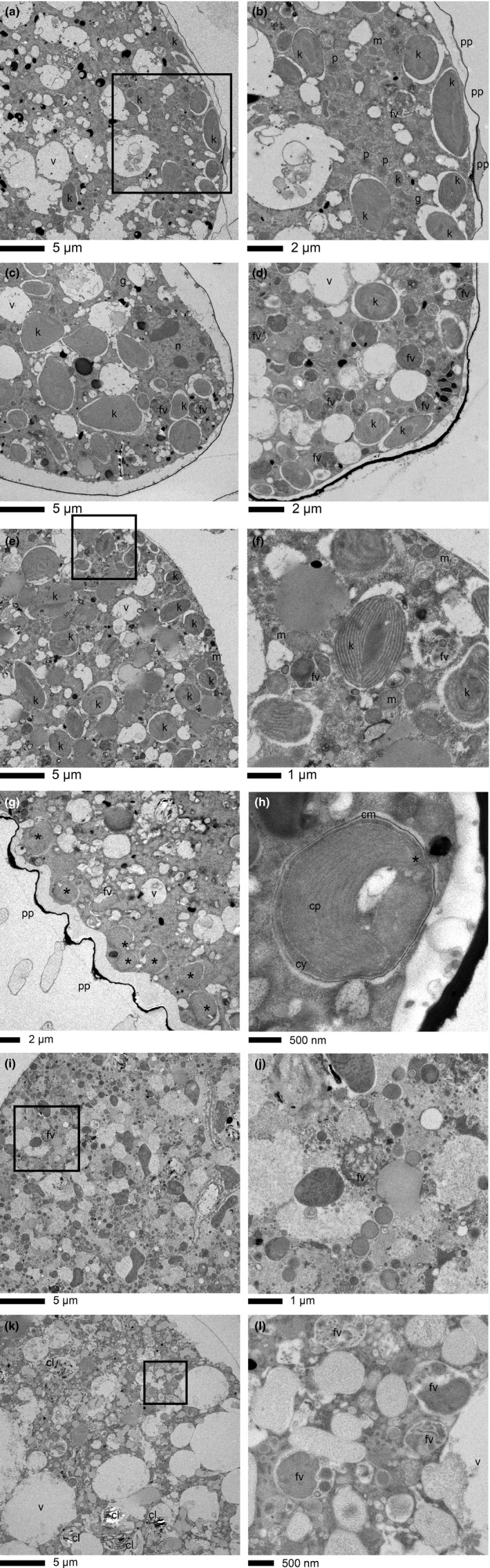
Transmission electron micrographs of rocky‐shore benthic foraminifera observed in this study. *Planoglabratella opercularis* (a and b), *Glabratella patelliformis* (c), *Angulodiscorbis quadrangularis* (d), and *Elphidium crispum* (e and f). Scale bar indicates 5 μm (a, c, and e), 2 μm (b and d), or 1 μm (f). k, kleptoplast; fv, food vacuole; v, vacuole; m, mitochondria; p, peroxisome; n, nucleus; and pp, pore plug. *Pararotalia nipponica* (g and h), *Cibicides lobatulus* (i and j), and *Quinqueloculina yabei* (k and l). Scale bar indicates 5 μm (i and k), 2 μm (g), 1 μm (j), or 500 nm (h and l). *endobiotic algae; fv, food vacuole; v, vacuole; cp, chloroplast; cm, cell membrane of the endobiotic algae; cy, cytoplasm of the endobiotic algae; pp, pore plug; and cl, clay mineral

The benthic foraminifera examined in this study contained different types of endobionts with different morphologies (Figure 1). Three species of the Glabratellidae, *P*. *opercularis*,* G*. *patelliformis*, and *A*. *quadrangularis*, harbored kleptoplasts from diatoms (Supporting information Table S1; Table 1; Figure 1a–d) (Jauffrais et al., 2018). *Planoglabratella opercularis* acquired chloroplasts from epiphytic diatoms, such as *G*. *patelliformis* and *A*. *quadrangularis* (Supporting information Table S1; Table 1). *Elphidium crispum* also acquired chloroplasts from epiphytic diatoms (Supporting information Table S1; Table 1; Figure 1e,f). Kleptoplasts derived from multiple species of diatoms, including not only marine species but also brackish and freshwater species, were found in some foraminifera, especially *P*. *opercularis*,*G*. *patelliformis*, and *E*. *crispum* (Table 1). We were able to identify some sequences at the species level using the *16S rRNA* gene. High sequence similarity (99%) suggested that kleptoplasts were derived from *Phaeodactylum tricornutum*,* Bacillaria paxillifer*,* Psammodictyon panduriforme*, and *Odontella sinensis*, which are marine species (Guiry & Guiry, 2018) (Table 1). However, other sequences could be identified only at the genus level, although the homology was high (97%–99%). Genera retained in foraminiferal cell cytoplasm as kleptoplasts included both *Amphora* sp. and *Navicula* sp., which are distributed in brackish waters, and *Pinnularia* sp., which is mainly distributed freshwater environments (Guiry & Guiry, 2018) (Table 1). The foraminifera were also found to contain numerous digestive residues in their food vacuoles (Figure 1a–f). We confirmed that kleptoplasts accumulated at the foraminiferal cell periphery in glabratellids, especially in *P*. *opercularis* (Figure 1a), whereas kleptoplasts spread throughout the entire cell cytoplasm in *E*. *crispum* (Figure 1e) (Jauffrais et al., 2018). In such cases, the foraminifera digest the diatom‐derived cytoplasm and organelles, but they can retain kleptoplasts and chloroplast‐like structures of captured microalgae in their food vacuoles. These findings suggest that these three glabratellid species and *E*. *crispum* have the potential to use kleptoplasts to acquire photosynthate and the remainder of the microalgae as food.

In contrast, *P*. *nipponica* had diatom endobionts in vacuoles (Figure 1g,h). The endobiotic *16S rRNA* gene sequence had high similarity to *Arcocellulus mammifer*, a marine species (Guiry & Guiry, 2018) (Table 1). Unlike kleptoplasts, these endobionts retain distinctive cell features, including the cell membrane, chloroplasts, and cytoplasm (Figure 1h). Digestive vacuoles were also visible in the *P*. *nipponica* (Figure 1g), *C*. *lobatulus* (Figure 1i), and *Q*. *yabei* (Figure 1k). However, no kleptoplast or endobiotic microalgae was found in *C*. *lobatulus* or *Q*. *yabei* based on TEM observations (Figure 1i–l) and genetic analyses. The same result was confirmed by the lack of amplification of the target plastid *16S rRNA* amplicons (Supporting information Table S1; Table 1).

We confirmed that there were digestive residues in the food vacuoles of these species (Figure 1j,l), although the food vacuoles of *Q*. *yabei* contained clay minerals (sediments; Figure 1k) with microalgal foods somewhat similar to residual food materials in the glabratellids and *E*. *crispum*. We could find no animal tissue (e.g., muscle tissue) as potential foods in any specimens, although in planktonic foraminifera, muscle tissue and its residues derived from copepod prey have frequently been identified by TEM (Caron & Bé, 1984; Hemleben, Spindler, & Anderson, 1989).

### 3.2 Nitrogen isotopic composition of amino acids and estimated TP_Glu/Phe_

**Figure 2:**
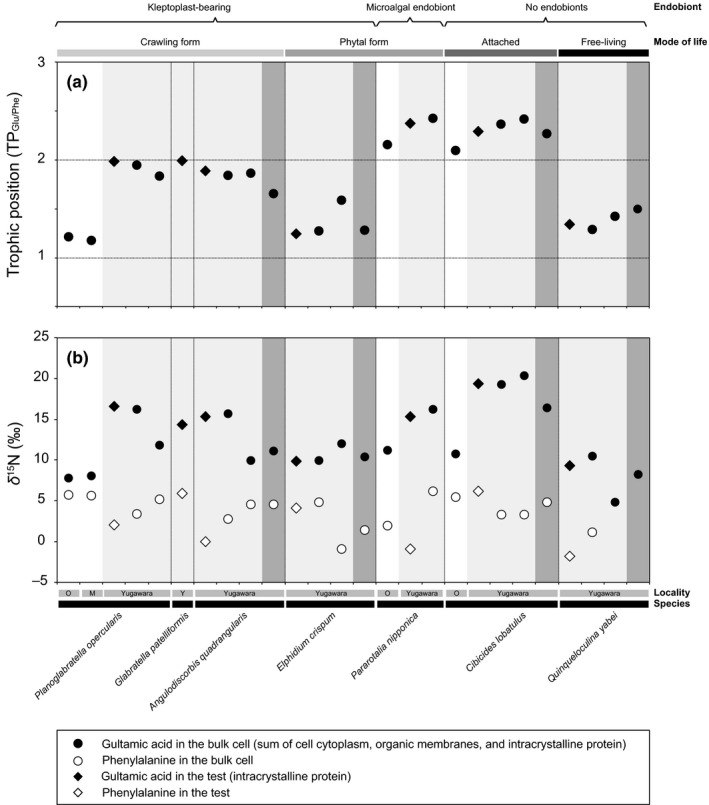
Estimated TP_G__lu/phe_ of rocky‐shore benthic foraminifera (a) and *δ* ^15^N values of glutamic acid (solid symbols) and phenylalanine (open symbols) (b). No shading (white area) indicates the samples collected in the daytime at the Omaezaki site for *P*. *opercularis*,* P*. *nipponica*, and *C*. *lobatulus* or at the Minami‐Izu site for *P*. *opercularis*; light gray indicates the samples that were collected in the daytime at the Yugawara site, and dark gray indicates the samples that were collected at night at the Yugawara site. Circles, bulk cell (including cell cytoplasm, organic membranes, intracrystalline proteins, endobionts, and food material); diamonds, and organic matter (including organic sheet and intracrystalline proteins in the test [shell]). Ecology of foraminifera including existence of endobionts and mode of life is indicated at the top of the panel, and locality and species name are indicated at the bottom of the panel

**Figure 3:**
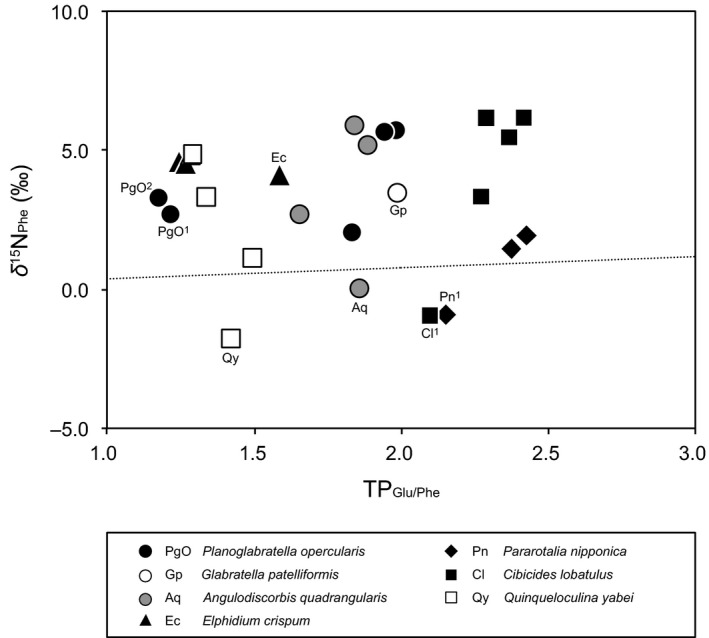
Relationship between TP_G__lu/Phe_ and nitrogen isotopic composition of phenylalanine (*δ* ^15^N _phe_). Dotted line indicates nitrogen isotope enrichment for phenylalanine (0.4‰) (Chikaraishi et al., 2009). The superscripts 1 and 2 indicate specimens collected at the Omaezaki and Minami‐Izu site, respectively. No superscript indicates samples collected at the Yugawara site

We found a large variation in the TP_Glu/Phe_ value among the seven species of rocky‐shore benthic foraminifera. The trophic positions seemed to depend on the different combinations of mode of life, food preference, and the morphological nature of the association between endobionts (Tables 1 and 2; Figures 2 and 3). In the case of kleptoplast‐bearing foraminifera, the TP_Glu/Phe_ values for Omaezaki and Minami‐Izu specimens of *P*. *opercularis* (TP = 1.2) were lower than the values for Yugawara specimens (1.9–2.0), whereas *E*. *crispum* had low TP_Glu/Phe_ values (1.2–1.6), even at Yugawara where received low light irradiances (Table 2; Figure 2a). Despite the fact that the kleptoplasts in both species originated from diatom chloroplasts (Supporting information Table S1; Figure 1), light intensities affected the TP_Glu/Phe_ values in *P*. *opercularis*, but not in *E*. *crispum* (Figure 2). In contrast, despite harboring algal endobioints from diatoms, the phytal form of *P*. *nipponica* exhibited high TP_Glu/Phe_ values (2.2 and 2.4) at both the Omaezaki and Yugawara sites. These TP values are similar to the TP_Glu/Phe_ values of an attached form of *C*. *lobatulus* without any kind of endobionts (2.1–2.4) at both locations (Figure 2a). The TP_Glu/Phe_ values of *P*. *nipponica* and *C*. *lobatulus* were very similar, regardless of light intensity. The TP_Glu/Phe_ values of both the kleptoplast‐bearing species and endobiont‐bearing species were slightly lower at night. *Quinqueloculina yabei*, classified as a free‐living form, exhibited TP_Glu/Phe_ values (1.3–1.5) as low as the values of *E*. *crispum* and *P. opercularis* (Figure 2a), although *Q. yabei* has no endobionts (Supporting information Table S1; Figure 1k,l).

There was much variation in the *δ* ^15^N value among species, as a function of light intensity, a result that primarily reflects the isotopic values of primary producers at the base of food webs (Table 2; Figures 2b and 3; Supporting information Figure S2a). We compared the *δ* ^15^N_Phe_ and TP_Glu/Phe_ values among *A*. *quadrangularis*,* E*. *crispum*,* C. lobatulus,* and *Q*. *yabei* between day and night. The *δ* ^15^N_Phe_ values were higher at night than during the day for *A*. *quadrangularis*,* E*. *crispum,* and *Q*. *yabei* (Table 2; Figure 3). However, the *δ* ^15^N_Glu_ value for *E*. *crispum* was lower at night than during the day, and the TP_Glu/Phe_ value was therefore high during the day in 2015. For both *E*. *crispum* and *Q*. *yabei*, the *δ* ^15^N values at night were similar to those collected in 2012. *Angulodiscorbis quadrangularis* had a larger TP_Glu/Phe_ value in the daytime; however, *Q*. *yabei* had almost the same TP_Glu/Phe_ value. Both the *δ* ^15^N_Phe_ and *δ* ^15^N_Glu_ values in *C*. *lobatulus* were lower at night than during the day. However, the corresponding TP_Glu/Phe_ values were identical. The* δ* ^15^N_Phe_ values were generally low in Omaezaki specimens, especially for *P*. *nipponica* (−1.9‰) and *C*. *lobatulus* (−1.9‰). They increased to 1.5‰–1.9‰ in *P*. *nipponica* and to 3.4‰ in *G*. *patelliformis*, and they exceeded 4.5‰ in other species that were collected in 2012 at Yugawara, including *P*. *opercularis* (5.6‰–5.7‰), *A*. *quadrangularis* (5.2‰–5.9‰), *E*. *crispum* (4.5‰–4.6‰), *C*. *lobatulus* (5.5‰–6.2‰), and the cytoplasm of *Q*. *yabei* (3.3‰–4.9‰). The values in 2015 were lower than those in 2012 at Yugawara, except for *E*. *crispum* (Tables 1 and 2; Figures 2b and 3). The trend of *δ* ^15^N_Glu_ was similar to that of *δ* ^15^N_Phe_ (Figure 2b; Supporting information Figure S2b). There were no differences in any species collected between the test and bulk cell *δ* ^15^N_Phe_ and TP_Glu/Phe_ values.

## 4 DISCUSSION

### 4.1 Microhabitat segregation and mode of life in relation to the trophic structure

**Figure 4:**
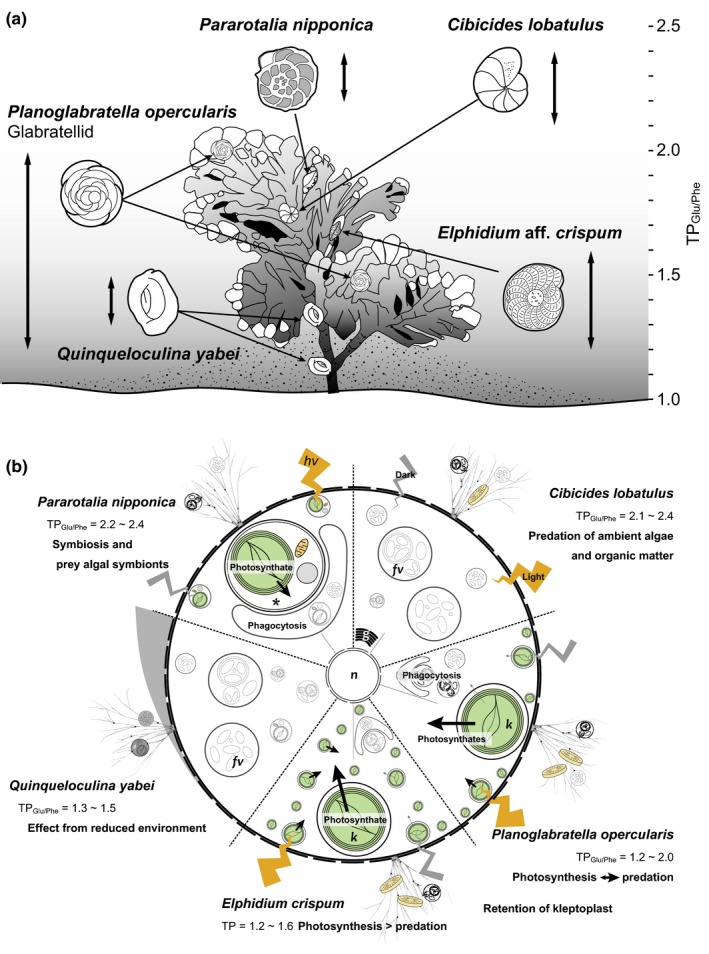
Schematic illustration indicating diverse trophic ecology in each species (a) and estimated resource usage of each species (b). Arrows indicate the life position of each foraminiferal species (modified from Kitazato, 1988). Double‐headed arrows indicate ranges of TP_G__lu/Phe_ values (a). Arrows indicate putative route of photosynthates and indicate differences in resource use between light and dark conditions (b). *endobiotic algae; k, kleptoplast; fv, food vacuole; n, nucleus; and g, Golgi apparatus

The estimated trophic positions of the rocky‐shore benthic foraminifera reflected their trophic hierarchy (Figure 4). These foraminifera had a large trophic diversity. The trophic position of only *C*. *lobatulus* was basically consistent with the ecology and food preference of this species relative to its mode of life based on previous culture experiment and/or field observations. This study resulted in five novel findings: (a) *P*. *nipponica* acquired its food mainly from preying on endobiotic diatoms and did not use photosynthate directly; (b) the low TP_Glu/Phe_ values in kleptoplast‐bearing *E*. *crispum* indicated that it depended mainly on photosynthates for nutrition, even though its microhabitat was characterized by low irradiances; (c) like *E*. *crispum*, the TP_Glu/Phe_ values of kleptoplast‐bearing *P*. *opercularis* were low, but only at high irradiances. At low irradiances, its TP_Glu/Phe_ values were high. The indication is that kleptoplast‐bearing *P*. *opercularis* used photosynthate directly from kleptoplasts in the former case and preyed on microalgae in the latter case; (d) *E*. *crispum* and *P*. *nipponica* had been thought to have a similar mode of life and to prey on exogenous microalgae in previous studies. However, their TP_Glu/Phe_ values were very different; (e) *Q*. *yabei* had low *δ* ^15^N and TP_Glu/Phe_ values, even though they did not contain any kind of endobiotic microalgae based on no amplification of PCR product and TEM observations.

Those trophic hierarchies provide evidence relevant to the morphology, the presence or absence of endobiotic microalgae, and food‐acquisition strategies of the foraminifera (Figure 2). These results clearly reveal that the foraminifera can use a wide range of nitrogen resources: photosynthates (for *E*. *crispum*), photosynthates + detritus (for glabratellids), predation of endobionts (for *P*. *nipponica*), and detritus (for *C*. *lobatulus*) (Figure 2). The TP_Glu/Phe_ values vary greatly within the algal microhabitat (Table 2). The large variations in the *δ* ^15^N_Phe_ value were consistent with trophic segregation among species, which in turn were influenced by locality and light intensities. We hypothesize that this trophic segregation enables a high biomass of foraminifera to live in diverse ecological niches within a microhabitat of coralline algae. Moreover, we found that the kleptoplast‐bearing foraminifera, especially *P*. *opercularis*, are mixotrophic; the trophic function is both primary producers and primary consumers in the studied environments. Kleptoplast‐bearing foraminifera use temporarily retained kleptoplastid photosynthates including amino acids, sugar, and lipid in general. The kleptoplast‐bearing foraminifera directly used at least nitrogen (amino acids) from the photosynthates, and hence, their TP_Glu/Phe_ values indicated a primary producer. In the following paragraphs, we compare our results of the trophic position with traditional scenarios suggested by previous studies.

The crawling form of the glabratellid species has kleptoplasts of diatom origin and intermediate TP_Glu/Phe_ values between primary producers and primary consumers (TP_Glu/Phe_ = 1.2 and 1.8–2.0) (Table 2; Figures 2a and 4a). *Planoglabratella opercularis* can change its trophic position as a function of light intensity in the coralline algae. In fact, the photosynthetic activity of this species, quantified in terms of oxygen production, rapidly responds to changes in light intensity (data not presented in this study). Glabratellid species, including *P*. *opercularis*, crawl on the thalli of coralline algae and are thought to graze on epiphytic microalgae—mainly diatoms—or organic detritus (Kitazato, 1988; Lipps, 1983). In fact, *P*. *opercularis* retains kleptoplasts of diatom origin that have been identified in other glabratellid species on the basis of TEM observations and blast homology searches (Supporting information Table S1; Table 1; Figure 1a–d). *Planoglabratella opercularis* can simultaneously use photosynthate and therefore is mixotrophic (Figure 4b). Foraminifera moved faster in epifaunal species than in infaunal species (Kitazato, 1988). Some foraminiferal species moved 1.9–5.5 times faster on the hard substrate such as glass surface than in the silty or sandy sediment, respectively, within a species (Kitazato, 1988). Thus, glabratellid species have the potential to migrate rapidly to suitable light conditions within a microhabitat to enable kleptoplast photosynthesis. They can simultaneously acquire microalgae as food when there is insufficient food available or light conditions become unfavorable (Figure 4a,b).

The phytal form of *E*. *crispum* had a TP_Glu/Phe_ value contributed from the primary‐producer mode (TP_Glu/Phe_ = 1.2–1.6) (Table 2; Figures 2a and 4a) and had kleptoplasts of diatom origin (Supporting information Table S1; Table 1; Figure 1e,f). This species lives suspended between the stems or thalli of coralline algae and expands its tough and thread‐like pseudopodia (granuloreticulopodia) to make a pseudopodial net (Figure 4a, Kitazato, 1988). *Elphidium crispum* can use its pseudopodial net to catch suspended organic material and small invertebrates. It is therefore thought to effectively acquire nutrition from organic material captured by pseudopodial nets in the space between the thalli and stem of coralline algae (Lipps, 1975, 1983). However, our results suggest that *E*. *crispum* uses photosynthates more than glabratellid species. *Elphidium crispum* can effectively use kleptoplastid photosynthates, and their kleptoplasts are more widely distributed in their cell cytoplasm than is the case with glabratellids (Figure 1e,f).

In contrast, the phytal form of *P*. *nipponica**,*** which has endobionts of diatom origin, has a higher position in the trophic hierarchy (TP_Glu/Phe_ = 2.2 and 2.4) (Table 2; Figures 2a and 4a) than *E*. *crispum* (Supporting information Table S1; Table 1; Figures 1g,h and 2). This difference suggests that *P*. *nipponica* preys on endobiotic diatoms and also on exogenous microalgae and higher‐trophic‐level organic matter (Figure 4b). A similar conclusion has been reached on the basis of TEM observations and culture experiments for *Baculogypsina sphaerulata* and *Calcarina gaudichaudii*, which bear diatom endobionts that they prey on (Röttger & Krüger, 1990), and for *Marginopora kudakajimaensis* and *Amphisorus hemprichii* (Lee et al., 1991), both of which are dinoflagellate‐bearing species. In the latter case, these species simultaneously use pseudopodial nets to entrap and prey on higher‐trophic‐level material, such as floating organic matter, or small invertebrates (Lipps, 1983). In the present study, we found that *P*. *nipponica* appeared to be a primary consumer; thus, endobiotic diatoms were food for this species.

The TP_Glu/Phe_ values of the attached, immobile form of *C*. *lobatulus* were high (2.1–2.4, Table 2; Figures 2 and 4a), a result consistent with previous studies (Kitazato, 1988; Langer, 1993). This species lacks endobiotic microalgae or kleptoplasts (Supporting information Table S1; Table 1; Figure 1i,j) and requires exogenous organic material (Figure 4b). In fact, we observed a number of food vacuoles containing digested residual material (Figure 1i,j). The fact that *C*. *lobatulus* attaches to a substrate with a secretion of organic glue suggests an epiphytic mode of life (Langer, 1993). This species spreads its pseudopodia along the surface of a substrate and into the overlying seawater to catch epiphytic microalgae; it feeds mainly on diatoms (Kitazato, 1988), floating organic material, or invertebrates (Kitazato, 1988; Langer, 1993).

The trophic position of the free‐living form of *Q*. *yabei*, a nonsymbiont‐bearing species, is enigmatic in that its TP_Glu/Phe_ value was low, 1.3–1.5 (Table 2; Figures 2 and 4a). The low *δ* ^15^N value of the organic matter in the porcellaneous calcite tests of *Q*. *yabei* was likely affected by the environmental conditions in the sediments. Its life position differs from that of other rocky‐shore benthic foraminifera, *Q*. *yabei* lives in and on the sediment trapped in the basal part of coralline algae (Kitazato, 1992). It is possible that environmental conditions in the pore water of the entrapped sediment surrounding *Q*. *yabei* affect its nitrogen isotopic composition (e.g., low *δ* ^15^N values of nitrate and/or ammonia in the pore water). The *δ* ^15^N_Phe_ values are actually lower than those of other species in the same habitat at the Yugawara site (Table 2; Supporting information Figure S2).

It is possible that the low *δ* ^15^N_Glu_ values in the bulk cell reflect the omnivorous behavior of *Q*. *yabei* (Kitazato, 1988, 1992; Myers, 1943; Turley, Gooday, & Green, 1993) (Supporting information Figure S2b; Figure 2). *Quinqueloculina yabei* scavenges the dead cells of other small protists and also preys on microbes (Kitazato, 1992; Muller & Lee, 1969) simultaneously retained with sediments (Figure 1k). A free‐living form of *Q*. *yabei* lives and moves in these reducing sediments; it preys on these microorganisms (Kitazato, 1988), takes up nitrate, and consumes sediments (Figure 1k,l) under low oxygen conditions. Therefore, the *δ* ^15^N values of *Q*. *yabei* may be affected by local environmental conditions and a combination of food sources. In contrast, high *δ* ^15^N values have been observed in cells of *Ammonia* sp. (phylotype T6, Hayward, Holzmann, Grenfell, Pawlowski, & Triggs, 2004), a shallow‐water (marsh) species probably affected by endobiotic microbes that they use intracellular nitrate pool (Nomaki et al., 2014), and in *Globobulimmina affinis*, a deep infaunal species found in bathyal sediments (Nomaki et al., 2015). The latter species is closely related to *G*. *pseudospinescens*, which is capable of complete denitrification; it accumulates intracellular nitrate and discharges as N_2_ (Risgaard‐Petersen et al., 2006). The low *δ* ^15^N values in *Q*. *yabei* are therefore not affected by endobiotic microbes or foraminiferal denitrification.

### 4.2 Effect of endobiotic microalgae on foraminiferal trophic function

The type and morphology of the endobiotic microalgae may affect the transport of organic materials (e.g., amino acids) between endobiont and host foraminiferal cytoplasm (Figure 4b). In fact, the trophic position of the kleptoplast‐bearing foraminifera varied at each sampling site as a function of the light availability, whereas endobiont‐bearing *P*. *nipponica* has consistently higher TP_Glu/Phe_ values (Table 2; Figures 2a and 4a). Indeed, the kleptoplast‐bearing rocky‐shore benthic foraminifera, especially for *E*. *crispum*, frequently use photosynthates from kleptoplasts directly as a nitrogen source and have TP_Glu/Phe_ values similar to those of photosynthetic organisms. In contrast, *P*. *nipponica* acquires nitrogen by predation on the endobiont; that is, there is a predator–prey interaction in terms of the amino acid metabolism between host and endobiont. The kleptoplast‐bearing foraminifera also behave as consumers in some situations, probably as a function of light intensity or availability of food, including the algal population around the foraminifera. In fact, the TP_Glu/Phe_ value (~1.2) for a specimen collected at Minami‐Izu and at Omaezaki suggests a primary producer at a high irradiance, whereas the TP_Glu/Phe_ value for specimens collected at Yugawara (1.8–2.0) indicates a primary consumer at a low irradiance (Table 2; Figure 2a). Furthermore, kleptoplast‐bearing foraminifera can maintain kleptoplasts functionally for certain periods without the kleptoplast's dividing (Correia & Lee, 2002; Grzymski, Schofield, Falkowski, & Bernhard, 2002; Jauffrais et al., 2016; Lopez, 1979). This behavior is advantageous from the standpoint of adaptation to a microhabitat, enables the coexistence of different species in a microhabitat. Therefore, foraminiferal species uses different nitrogen sources in a habitat and thus maintain high biomass of low trophic‐level organisms per unit area.

The photosynthetic activity of kleptoplasts clearly affects the host foraminiferal trophic requirements. Intertidal, shallow‐water benthic foraminifera must frequently retain new kleptoplasts; functional kleptoplasts are potentially retained for up to 21 days in *Haynesina germanica* and up to 11 days in *Ammonia tepida* in the dark (Jauffrais et al., 2016). Retention times are shorter (about 1 week) in the light (Jauffrais et al., 2016). The glabratellids observed in this study retain kleptoplasts of diatom origin and display mixotrophic behavior (Supporting information Table S1; Table 1, Figure 2a,b). *Planoglabratella opercularis*, for example, retained kleptoplasts longer in the dark than in the light (data not presented in this study). In the case of the sea slug *Plakobranchus ocellatus*, Maeda et al. (2012) found that the photosynthates of kleptoplasts do not contribute to the nutrition of wild specimens (TP_Glu/Phe_ = 1.9), whereas starved specimens in the laboratory rely largely on photosynthates (TP_Glu/Phe_ = 1.3). They have proposed that *P*. *ocellatus* acquires kleptoplasts for complementary nutrition under starved conditions. Unlike the sea slug, the kleptoplast‐bearing foraminifer *P*. *opercularis* changes its trophic (nitrogen) source from food to photosynthates as a function of irradiance. *Elphidium crispum* depends on photosynthates from kleptoplasts of diatom origin that it retains, as do glabratellids, although its TP_Glu/Phe_ value was lower than that of glabratellids within the same microhabitat at the Yugawara site (TP_Glu/Phe_ = 1.2, 1.3) (Table 2; Figure 2). The distribution of kleptoplasts in the cell differs between glabratellids and *E*. *crispum* (Figure 1). The kleptoplasts are distributed at the periphery of the cell just beneath the pore plug in the former species, whereas they are distributed densely in the endoplasm in the latter species (Figure 1a–f) (Jauffrais et al., 2018). These differences may affect photosynthetic efficiency.

Although *C*. *lobatulus* and *P*. *nipponica* had similar TP_Glu/Phe_ values (TP_Glu/Phe_ = 2.1–2.4), the sources of their foods differed (Table 2; Figures 2,3 and 4b). *Cibicides lobatulus* used neither endobiotic microalgae nor kleptoplasts (Supporting information Table S1; Table 1; Figure 1i,j), Instead, the TP_Glu/Phe_ value was consistent with previous observations that *C*. *lobatulus* preys on epiphytic microalgae and floating organic material or invertebrates (Kitazato, 1988; Langer, 1993). In contrast, *P*. *nipponica* is thought to use photosynthates from “symbionts”; however, its TP_Glu/Phe_ values suggest that this species depends mainly on predation of endobionts as a nitrogen source. It is possible that the endobionts in *P*. *nipponica* assimilate nitrogen from both the ambient environments and from the host foraminifer; therefore, its *δ* ^15^N value is lower than that of *C*. *lobatulus* (Table 2; Figures 2 and 3). The *δ* ^15^N values of *P*. *nipponica* also differ from those of the kleptoplast‐bearing glabratellids. The significant difference between the *δ* ^15^N values of these species (Table 2) suggests that *P*. *nipponica* cannot directly use photosynthates as a nitrogen source.

In an interesting manner, multiple nitrogen sources potentially exist in a microhabitat. The fact that rocky‐shore benthic foraminifera with the same TP_Glu/Phe_ values have distinct *δ* ^15^N_Phe_ values suggests that the nitrogen sources they exploit in their microhabitats vary as a function of irradiance and food availability (Table 2; Figures 2 and 3). The phytal and crawling forms, glabratellids and *E*. *crispum*, respectively, retain kleptoplasts but have a different mode of life; their trophic requirements therefore change as a function of their location in the coralline algae, irradiance (as affected by seaweed density), and microtopography. It is highly possible that the rocky‐shore benthic foraminifera assimilate these nitrogen sources (Figure 3). These observations suggest that each of these species of foraminifera uses a different source of nitrogen and hence imply microhabitat (niche) segregation.

In benthic foraminifera, the maximum cell density is often several hundred to several thousand individuals 10 cm^2^ within a microhabitat (e.g. Murray, 2006). For example, Kitazato (1986) found that the density of benthic foraminifera ranged from 158 to 408 specimens 10 cm^2^ on the coralline algae on an intertidal rocky shore. To maintain such high densities of individual specimens within a microhabitat, it is important that the foraminifera use multiple nitrogen sources or vary their trophic hierarchy, as do kleptoplast‐bearing foraminifera or microbial associations (Nomaki et al., 2015).

It should be noted that our results are a snapshot in time that reflects different environmental conditions. However, at the same time, the observed TP_Glu/Phe_ values and *δ* ^15^N values were averages based on 10–200 specimens of each foraminifer (Table 1). It is noteworthy that each measured value was significantly different from the others and was characterized by a low propagation error (Table 2; Figure 2). To clarify any microhabitat niche segregation in terms of resource use, further detailed observations will be needed to determine whether the trophic hierarchy of kleptoplast‐bearing species changes as a function of irradiance or the source of amino acid nitrogen.

## 5 IMPLICATIONS

Rocky‐shore benthic foraminiferal species can adapt, coexist, and maintain their high abundance and biomass within a microhabitat of coralline algae by utilizing multiple nitrogen resources and their trophic requirements. The present study has shown that diverse trophic ecology can be demonstrated via compound‐specific stable nitrogen isotope analysis of amino acids. Foraminifera prey on not only epiphytic diatoms that flourish on the coralline algae but also on exogenous organic material and on endobiotic microalgae. Some species of kleptoplast‐bearing foraminifera can use endobiotic algae to obtain photosynthates and also can prey on exogenous microalgae. It is possible that this mixotrophic behavior is one of the adaptation mechanisms that accounts for the high biomass and complex microtopography of the intertidal rocky shore. The mode of life of foraminiferal species indicates behavioral similarities but does not evidence similarity of use (predation) food materials. Mixotrophy, as seen in glabratellids, provides an advantage for nutrition acquisition to kleptoplast‐bearing foraminifera; they use the photosynthates from kleptoplasts when there is insufficient food because of seasonal variations in food supply and competition with other species. They also acquire food through predation on ambient microalgae when kleptoplasts cannot provide sufficient photosynthates because of a deficiency in light due to weather or microtopography.

In recent decades, mixotrophic protists have been found in many taxonomic groups that play important roles in energy flow and biogeochemical cycling in ecosystems. These roles have been revealed by computer simulation models (e.g., Mitra et al., 2014, 2016; Stocker, 1998) and analysis of gene expression in the case of mixotrophic protists (Liu, Campbell, Heidelberg, & Caron, 2016). Mixotrophic strategies become an even greater factor in an ecosystem from the standpoint of nutritional strategies and physiology. The mixotrophic strategies of kleptoplast‐bearing foraminifera and microhabitat preferences of foraminifera apparent in this study can be an integral part of a marine ecosystem and reveal the physiological mechanisms and nutritional strategies of mixotrophs.

From another perspective, the accurate trophic position determination based on compound‐specific nitrogen isotope analysis can elucidate the trophic requirement for diverse genotypes in cryptic species within a foraminiferal morphospecies (e.g., Tsuchiya et al., 2014). We suggest that ecological factors such as the different trophic requirements of each foraminiferal species can contribute microhabitat segregation in a habitat within the range of foraminiferal movement. These ecological factors can provoke sympatric cryptic speciation of foraminifera.

## CONFLICT OF INTEREST

None declared.

## AUTHOR CONTRIBUTIONS

Conceived and designed the experiments: M Tsuchiya. Performed the experiments for nitrogen isotope analysis: M Tsuchiya, Y Chikaraishi, and Y Sasaki. Performed the experiments for TEM observation: M Tsuchiya, H Nomaki, A Tame, and K Uematsu. Performed the experiments for molecular work: M Tsuchiya. Analyzed the data: M Tsuchiya, Y Chikaraishi, H Nomaki, Y Sasaki, and N Ohkouchi. Prepared the first draft: M Tsuchiya, Y Chikaraishi, H Nomaki, and N Ohkouchi. Revised the manuscript: Y Sasaki, A Tame, and K Uematsu. All authors participated in discussions.

## DATA ACCESSIBILITY

DNA sequences: GenBank accessions KY498705‐KY498734.

## Supporting information


** **

** **

** **
