# Supplementary material for: Compound‐specific isotope analysis of benthic foraminifer amino acids suggests microhabitat variability in rocky‐shore environments
Source: Ecol Evol. 2018 Jul 24;8(16):8380–95. doi: 10.1002/ece3.4358 (PMC6144965; doi:10.1002/ece3.4358)
Supplement: Supplementary file 3 [file ECE3-8-8380-s003.docx]

**TABLE S1** BLAST homology search results for host nuclear *rRNA* gene and endobiont plastid *16S rRNA* gene in each species.

| Species | Sample ID | Closest sequence | (accession number) | Origin of  symbiont^1^ | Gene^2^ | Number of clones | Coverage  (%) | Homology  (%) | Accession number^3^ |
| --- | --- | --- | --- | --- | --- | --- | --- | --- | --- |
| *Planoglabratella* *opercularis* | 09 | *P*. *opercularis* | (AF498340) | F | I | 3 | 100 | 99 | *KP792494*  *KP792495*  *KP792496* |
|  | 09 | *Phaeodactylum* *tricornutum* | (EF067920) | B | P | 2 | 100 | 97 | *KP792453*  *KP792454* |
|  | 09 | *Pinnularia* sp. | (KT952293) | B | P | 1 | 100 | 97 | *KP792455* |
| *Glabratella* *patelliformis* | 446 | *G*. *patelliformis* | (AF194078) | F | S | 3 | 100 | 99 | **KY498714**  **KY498715**  **KY498716** |
|  | 446 | *Amphora* sp. | (FJ002217) | B | P | 1 | 100 | 99 | **KY498705** |
|  | 446 | *Navicula* sp. | (FJ002226) | B | P | 1 | 100 | 99 | **KY498706** |
|  | 446 | *Bacillaria* *paxillifer* | (AJ536452) | B | P | 1 | 100 | 99 | **KY498707** |
|  | 446 | *Psammodictyon* *panduriforme* | (FJ002157) | B | P | 1 | 100 | 99 | **KY498708** |
| *Angulodiscorbis* *quadrangularis* | 159 | *A*. *quadrangularis* |  | F | S | 1 |  |  | *AF194076* |
|  | 159 | *Navicula* sp. | (FJ002226) | B | P | 1 | 100 | 99 | **KY498709** |
| *Elphidium* *crispum* | 956 | *Elphidium* *macellum* | (JN655700) | F | S | 2 | 100 | 86 | **KY498717**  **KY498718** |
|  | 956 | *Elphidium* *margaritaceum* | (JN655700) | F | S | 1 | 100 | 86 | **KY498719** |
|  | 956 | *Odontella* *sinensis* | (Z67753) | B | P | 1 | 100 | 99 | **KY498710** |
|  | 965 | *Elphidium* *macellum* | (JN655700) | F | S | 3 | 100 | 86 | **KY498720**  **KY498721**  **KY498722** |
|  | 965 | *Pinnularia* sp. | (KT952293) | B | P | 1 | 100 | 96 | **KY498711** |
| *Pararotalia* *nipponica* | 961 | *P*. *nipponica* | (AJ879137) | F | S | 3 | 100 | 99 | **KY498723**  **KY498724**  **KY498725** |
|  | 961 | *Arcocellulus* *mammifer* | (FJ002193) | B | P | 1 | 100 | 99 | **KY498712** |
|  | 967 | *P*. *nipponica* | (AJ879137) | F | S | 3 | 100 | 99 | **KY498726**  **KY498727**  **KY498728** |
|  | 967 | *Arcocellulus* *mammifer* | (FJ002193) | B | P | 1 | 100 | 99 | **KY498713** |
| *Cibicides* *lobatulus* | 970 | *C*. *lobatulus* | (AY934742) | F | S | 3 | 100 | 97 | **KY498729**  **KY498730**  **KY498731** |
| *Quinqueloculina* *yabei* | 971 | *Q*. *seminulum* | (HE998688) | F | S | 3 | 98 | 95 | **KY498732**  **KY498733**  **KY498734** |

Shaded areas indicate the closest sequences for endobiots.

^1^B, Bacillariophyta (diatom); F, Foraminifera

^2^I, Internal transcribed spacers (ITS) of nuclear ribosomal RNA (*rRNA*); P, plastid *16S rRNA*; S, nuclear small subunit (*18S*) *rRNA*.

^3^Data available in genbank. Accession numbers of new data are shown in bold, previous data are shown in italic.
